# Towards understanding and improving medication safety for patients with mental illness in primary care: A multimethod study

**DOI:** 10.1111/hex.14095

**Published:** 2024-05-30

**Authors:** Matthew J. Ayre, Penny J. Lewis, Denham L. Phipps, Kathy M. Morgan, Richard N. Keers

**Affiliations:** ^1^ Division of Pharmacy and Optometry, Faculty of Biology, Medicine and Health, School of Health Sciences The University of Manchester Manchester UK; ^2^ NIHR Greater Manchester Patient Safety Research Collaboration, Manchester Academic Health Science Centre (MAHSC) The University of Manchester Manchester UK; ^3^ Manchester University NHS Foundation Trust Manchester UK; ^4^ Pharmacy Department Pennine Care NHS Foundation Trust Manchester UK; ^5^ Optimising Outcomes With Medicines (OptiMed) Research Unit Pennine Care NHS Foundation Trust Manchester UK

**Keywords:** community mental health services, holistic mental healthcare, intervention, medication safety, primary health care, psychiatry, shared decision‐making

## Abstract

**Introduction:**

Medication safety incidents have been identified as an important target to improve patient safety in mental healthcare. Despite this, the causes of preventable medication safety incidents affecting patients with mental illness have historically been poorly understood, with research now addressing this knowledge gap through a healthcare professional lens. However, patients and carers can also provide complimentary insight into safety issues, and as key stakeholders in healthcare, it is vital to consider their needs when designing effective interventions.

**Methods:**

A two‐stage approach was adopted by (i) conducting three focus groups (FG) comprising 13 patients with mental illness and their carers to develop a holistic picture of medication safety in primary care with extraction of themes guided by the P‐MEDS framework; (ii) conducting two separate nominal group consensus workshops with seven patients with mental illness/carers and seven healthcare professionals to identify priority areas for targeted interventions.

**Results:**

Seven themes were identified in the FGs: communication; trust, involvement and respect; continuity and support; access; the healthcare professional; the patient and carer; and the organisation. Priority areas identified for intervention by key stakeholders included improving communication within and between clinical services, enhancing patient support with holistic continuity of care, maximising shared decision‐making and empowerment, ensuring timely access to medicines and services, strengthening healthcare professional knowledge regarding mental illnesses and associated medications, and increasing patient dignity and respect.

**Conclusion:**

This study has developed a holistic picture of contributors to medication safety incidents affecting patients with mental illness in primary care. This theory was then used by key stakeholders to inform and generate priority recommendations for targeted interventions. These findings can be used to inform future intervention research, as they consider the needs of those who access or work within primary care services.

**Patient or Public Contribution:**

An advisory group consisting of three expert patients with lived experience of mental illness was consulted on the design of both stages of this study. Patients with mental illness and/or their carers were recruited and participated in both stages of this study. Patients/carers aided with data analysis and interpretation during the patient/carer nominal group consensus workshop.

## 1 INTRODUCTION

Both patient and medication safety in psychiatry are key targets for improvement within healthcare systems worldwide.^1^, ^2^, ^3^, ^4^, ^5^ In England, an estimated 237 million medication errors occur each year with a significant proportion (38.4%) originating in primary care settings, which can lead to hospital admissions and increased healthcare costs.^6^, ^7^ In the United Kingdom (UK), mental health services can be delivered from primary, secondary or tertiary care^8^ with 90% of patients with mental illness receiving treatment solely in primary care settings.^9^ The National Health Service Long Term Plan published in 2019 proposed integration of community mental health services, comprised of multidisciplinary teams supporting patients with more complex needs within the community.^2^, ^8^ A recent scoping review outlined that patients with mental illness experience various medication safety challenges in primary care, some being inherently preventable.^10^ However, the majority of studies investigating causes of these challenges focus on nonadherence, with the causes of preventable medication safety incidents being poorly understood,^10^ which limits the development of effective interventions.^4^, ^5^

A recent exploratory study with UK‐based healthcare professionals provided some initial insight into potential contributory factors leading to preventable medication safety incidents affecting this patient group.^11^ Patients and carers, though, also play a crucial role in medication and patient safety and their perspectives can further improve understanding of medication safety issues, as well as often identifying safety factors which may differ to those identified by clinicians.^12^, ^13^, ^14^, ^15^, ^16^, ^17^, ^18^, ^19^ Patients and carers are able to provide complementary views on medication safety, as well as themselves being important barriers to preventable harm.^20^ Addressing factors which they consider to be important, as well as actively involving them in improvement, can enhance the effectiveness of improvement interventions.^21^, ^22^, ^23^, ^24^ Therefore, as key stakeholders in healthcare, it is important that their needs are considered when devising interventions to improve medication safety.^15^, ^23^, ^24^, ^25^ By first creating a holistic picture of this topic, this theory can be utilised to design patient safety interventions which will help demonstrate feasibility and acceptability.^26^

The aims of this multimethod study were therefore to (i) elicit patient/carer perspectives on mental health medication safety in primary care; and to (ii) generate stakeholder‐led recommendations (from healthcare professionals, patients and carers) for improving medication safety for patients with mental illness in primary care.

## 2 MATERIALS AND METHODS

This multimethod study was conducted in two stages. First, qualitative focus groups (FG) were conducted to investigate patients and carers perceptions of safety in relation to medication in primary care. Second, the findings from these FGs, as well as from previous research,^11^ informed stakeholder‐led consensus workshops to identify and prioritise areas for intervention.

### 2.1 Participant selection

For stage 1, the sampling frame comprised patients with mental illness and carers of patients with mental illness. For stage 2, the sampling frame comprised patients with mental illness, carers and primary care‐based healthcare professionals. Within each frame, participants were identified by purposive sampling using specific inclusion criteria, which specified that participants must have either accessed or worked within primary care services within the last 5 years. This ensured they would be able to provide detailed information relevant to the study question,^27^ as they would be able to discuss risks with medication for this patient group.^28^, ^29^, ^30^ Participants who met the eligibility criteria were then enrolled according to their availability and accessibility.^31^

### 2.2 Patient and public involvement and engagement

The study design, FG schedule and consensus workshop schedule were discussed with a patient group consisting of three members (two females and one male) with lived experience of mental illness and using health services. Their feedback was used to refine the FG and workshop schedules, as well as contribute to the decision to conduct split consensus workshops with patients/carers and healthcare professionals.

### 2.3 Recruitment

Participants for stages 1 and 2 were recruited via distribution of an advertisement email and flyer amongst regional and national contacts within the research team, participants who had consented to future research from previous studies, NIHR Research for the Future (https://www.researchforthefuture.org/) and relevant social media channels with direct links to mental health support groups. All participants gave informed consent before taking part and were reimbursed with an electronic shopping voucher after participation.

### 2.4 Data collection

Both stage 1 FGs and stage 2 consensus workshops were conducted online using Zoom™. The stage 1 FGs were scheduled to last ∼2 h^32^ with stage 2 workshops lasting ∼2.5 h including sufficient breaks.^33^

In stage 1, each FG session was run by two facilitators: M. J. A. and another member of the research team (R. N. K., P. J. L. or D. L. P.). The FG discussion, guided by a schedule (File S1), began by asking participants to describe what made them feel safe or unsafe with their medicines outside of the hospital. They were then asked to describe how these things led to them feeling safe or unsafe, and what might need to change for them to feel safer with their medicines outside of hospital. The findings from these FGs were combined with a healthcare professional perspective data set ^11^ to present to participants in the stage 2 consensus workshops.

**Figure 1:**
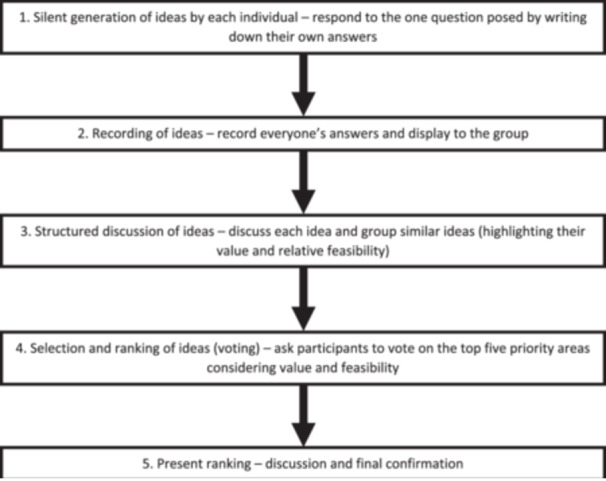
Nominal group technique process. Adapted figure from McMillan et al.^36^

Stage 2 consensus workshops were conducted with patients/carers and healthcare professionals separately. This design was adopted to mitigate the power imbalance which has been noted between clinicians and patients with mental illness.^11^, ^34^, ^35^ Both workshops were moderated by two facilitators; M. J. A. facilitated both workshops with either R. N. K. or P. J. L. co‐facilitating a workshop. The question used to initiate nominal group discussion was: “What do you think are the most important areas that could be changed to help improve medication safety for patients with mental illness outside of hospital?” The workshop followed a schedule guided by the nominal group technique^36^ and a detailed outline of the process can be viewed in Figure 1. The combined dataset was presented to the participants before commencing the workshop, which could then be used to help inform their ideas around which areas may be amenable to intervention. The first workshop was conducted with patients/carers, and their anonymised rankings were presented in the second workshop involving healthcare professionals. This was done to help ensure the research was patient/carer led and healthcare professionals could then consider the needs of patients/carers during the idea generation, discussion and voting stages. Between each stage of the nominal group discussion, there was a break to allow the researchers to organise the ideas generated into generic themes based on keywords. Participants subsequently discussed, grouped and named the themes.

All FG sessions and consensus workshops were audio recorded and professionally transcribed. Returned transcripts were checked and verified against the original recordings by M. J. A. The FG discussion durations ranged between 92 and 95 min and both consensus workshops lasted approximately 150 min.

### 2.5 Data analysis

Stage 1 FG data were thematically analysed using the framework method.^37^ All transcripts were coded independently by M. J. A, who then organised the data into a framework based on the P‐MEDS framework.^38^ This framework has been devised to examine the contributory factors of medication safety incidents from a patient perspective. An inductive approach to analysis was adopted as the framework provided an initial set of themes which could be developed throughout the analysis. All of the transcripts were read and coded by the remainder of the research team (R. N. K., P. J. L., D. L. P. and K. M. M.) and consultation was carried out to reach agreement on the themes.

For stage 2 workshops, the number of themes available to vote on depends on the research topic, however, five is typically common in nominal group activity.^39^, ^40^ Five themes were generated by the participants in both consensus workshops after completion of step three (see Figure 1), and these areas were assigned points, with the highest priority scoring five and the lowest priority scoring one.^36^ Participants were then asked to cast their votes simultaneously, using the chat function in Zoom™, for the highest priority followed by the second highest and so forth. The points for each theme were then added up to provide the nominal group technique score to produce the final ranking.

### 2.6 Ethical issues

All participants provided informed written consent to participate in either the stage 1 FG discussions or the stage 2 consensus workshops. Each participant was assigned a pseudonymous identifier to maintain confidentiality.

## 3 RESULTS

### 3.1 Stage 1: FGs

Data were collected via three FGs with 13 participants in total (FG1 *n* = 4, FG2 *n* = 4, FG3 *n*= 5). Out of the 13 participants, 11 were those with lived experience and 6 were carers (4 participants had both roles). There was representation across all age range brackets, with female participants representing 61.5% of the total sample. Not all ethnic groups were represented, the majority of participants (*n* = 9/13) being White British. Table 1 provides a full breakdown of the FG participants.

**Table 1:** Focus group participants.

Focus group number	Patient/carer ID	Age range	Sex (M/F)	Ethnicity
1	Carer 01	18–24	F	White British
	Carer 02	55–64	M	Asian Indian
	Patient 03	45–54	F	White British
	Patient 04	35–44	M	White British
2	Patient 05	25–34	F	White British
	Patient 06	25–34	M	White British
	Patient/carer 07	55–64	M	White British
	Patient 08	55–64	F	White—any other background
3	Patient 09	35–44	F	White British
	Patient/carer 10	45–54	F	Mixed—any other background
	Patient/carer 11	35–44	M	White British
	Patient/carer 12	65+	F	White British
	Patient 13	65+	F	NS

Abbreviations: F, female; M, male; NS, nonspecified (participant preferred not to say).

### 3.2 Contributory factors to feeling unsafe/safe with medication

#### 3.2.1 Communication

Communication between professionals was highlighted by participants as a key influence on their feelings of safety with their medication (see File S2 for additional quotes regarding the FG themes). It was reported that different healthcare records and clinical letters may have incomplete or inconsistent clinical information such as treatment details. Not communicating the necessary clinical information was described by participants to result in some clinicians not having a clear clinical picture of the patient, which could lead to the receipt of duplicate prescriptions and contraindicated medication. Communication between professionals and patients was also considered to be important, as participants reported receiving inadequate information regarding their medications, little reassurance about medication risks, unsatisfactory responses to medication‐related queries and contradictory information from healthcare professionals. For example, one participant described receiving contradictory advice from different general practitioners (GP) about the required dose of a psychotropic:

One [GP] was saying take X dose, and the other one [GP] was saying take this dose. […] And we ended up saying, well which one is it? And then we had to go and speak to somebody else and then for them to say, oh give this […] and we had to go with the majority basically. (Carer 01, FG1)

#### 3.2.2 Trust, involvement and respect

Participants described a lack of empowerment when it came to decisions about their treatment. Professionals were reported to sometimes change or add medication without consultation with the patient and were reluctant to engage in discussions when the patient raised concerns. During consultations, participants likened the experience to feeling “like a child” (Patient/carer 07, FG2) as they were expected to simply comply with the medication regimen without any input into their treatment, as outlined by one patient:

if you tried to bring things up, […] because you're not the professional in the situation, it's not always taken into account what your wishes are. But then […] you're expected to manage your own medications and take all your medications and then […] recount about what you've said to other GPs or other health professionals. And you're […] expected to be responsible for it all. But then you're not as involved in the decisions about your own medications. (Patient 06, FG2)

Healthcare professional behaviour and attitude during consultations were seen to contribute to patients feeling unsafe. Participants reported professionals lacking empathy, not encouraging deeper discussions, advocating medication above other treatment options that participants wished to explore and refusing to change medicines when patients/carers raised concerns. Stigmatising behaviours and attitudes of healthcare professionals were reported by participants who felt that they were treated unfairly compared to other patient groups without mental illness in regard to lack of involvement in treatment decisions and the manners displayed in the clinician consultation. Ultimately, participants reported an imbalance of power within the healthcare professional–patient relationship, a lack of involvement in decision‐making and a need to take matters into their own hands because professionals did not have open conversations with them about therapeutic options. As a result, participants felt that advocating about their medication was not effective and that professionals did not dedicate sufficient attention to what they felt were important issues raised during their consultations, since “they're just another one with mental health issues” (Patient 05, FG2).

#### 3.2.3 Continuity and support

Participants reported how they could be left to take medication without a follow‐up review in place to discuss efficacy and side effects. Participants would then have to take it upon themselves to initiate such follow‐up appointments, which could be a challenge during periods of illness. They described how regular follow‐up appointments created comfort and security; on the other hand, it was felt that being left to endure treatments that may not be entirely suitable for them without any clinician oversight increased feelings of risk. A reported lack of continuity between the differing healthcare professionals they encountered made them feel less safe with their medication. In particular, participants reported becoming distressed at having to repeat the same information to different professionals and that some healthcare professionals may not know them well enough to know what works for them, provoking anxiety about the prescribing/changing of medication. Conversely, consultations with a known and familiar professional were reported to increase feelings of trust and safety, as highlighted by one patient:

…there's so many professionals involved that they just don't know me. I like to have at least […] one or two people who generally know me over the years and then I've got more trust in them that they know what's going to work well for me. Like, things that have worked in the past, that didn't work. Yeah, they just know what's healthy behaviour for me and what's signs of me going downhill. (Patient 03, FG1)

Participants found different information resources helpful for answering any queries or concerns they may have had about their medication such as official health advice websites, forums and mental health charities. Professional clinical resources and unofficial websites (lacking a recognised logo) were of concern and were considered to increase feelings of anxiety about medication. However, participants reported not always knowing which resources to use or where to find the information they needed. Sometimes information could be confusing or create fear regarding the medication, which could contribute to reluctance to take it as prescribed.

#### 3.2.4 Access

Participants described timely access to medication that was the correct strength, dose, in a safe quantity and with minimal drug–drug interactions as an important factor in feeling safe. Respondents discussed that there could sometimes be delays between general practices sending prescriptions to pharmacies and medication being out of stock, which could increase anxiety, and in some cases worsen symptoms or cause medication withdrawal. Participants described difficulties/barriers to accessing services and seeing healthcare professionals to help resolve issues, particularly during out‐of‐hours or weekends. They highlighted that different appointment formats could create anxieties. On the one hand, they felt clinicians cannot respond to physical cues remotely; but on the other hand, there may be travel difficulties in attending clinics physically. Ultimately, access to individualised care was viewed as an important factor, since participants reported personalised consultations regarding their medication as a positive factor for increasing feelings of safety. Being met with barriers to accessing timely personalised care was reported to increase participants' feelings of risk with their medication, as one patient highlighted:

My mental health team is not in my local area. I have to go out of area. So that's stressful as well and the waiting about, whether it's actually going to be there or you can turn up and then the person was off sick, so I've still not got it. And it's that … so you're trying really hard to help yourself and be as independent as possible and it's almost like you're met with barriers all the time. (Patient 03, FG1)

#### 3.2.5 The healthcare professional

Participants described how they felt some prescribers lacked knowledge regarding medication for mental illness and associated medications, impacting the perceived ability to prescribe safely. This lack of confidence in healthcare professionals led to patients feeling unsafe when such prescribers suggested a prescription, as outlined by one participant:

I have experienced where the GP has prescribed something and I've just had to cross my fingers that he knew what he was doing… (Patient/carer 12, FG3)

On the other hand, when professionals were perceived to demonstrate competence when answering patients'/carers' queries regarding psychotropic medication, this increased feelings of safety.

#### 3.2.6 The patient and carer

Participants described that they had a lack of knowledge/understanding regarding medication and healthcare professional roles. Participants were more comfortable with clinicians who were qualified to carry out additional clinical tasks such as prescribing medication as it enhanced the clinicians' competence in the eyes of the patient. When participants were left confused (as mentioned earlier) and resulted to searching for answers themselves, it made them feel unsafe relying on their own knowledge and judgement. Participants described how they felt the responsibility for managing their medication(s) was being placed on them more often over time due to resource constraints. Respondents described how their prescribers did not support them in the management of their medication and how this may force patients/carers to take charge of their own treatment, which could sometimes be a positive step towards empowerment for some patients and carers. However, participants reported that this could also result in patients/carers taking more decisions which were felt to sometimes carry risk, especially if they were less well informed. One patient described how they decided to taper off their medication themselves as they felt the prescriber provided few options for alternative discussion:

I've come off my medications before and I ended up doing it without talking to the GP about it ‘cause I felt like they wanted me to stay on it. (Patient 06, FG2)

A minority of participants discussed that as a patient's mental health condition status may fluctuate and during periods of illness, it can become challenging to communicate care needs appropriately. This could be particularly challenging for participants when, for example, unexpected adverse drug reactions occurred and required hospital interventions, and the seriousness of these reactions then creates ongoing concerns that the treatment may carry increased risk.

#### 3.2.7 The organisation

The structure of healthcare services, with its differing teams of healthcare professionals often working in silos, was reported by participants to contribute to feeling unsafe with their medication. Participants reported how they wanted to see the right professionals at the right time, and that the service structure inhibited that from happening, as described earlier regarding continuity and access. It was reported how, once they accessed a service, they may see professionals who cannot help with their healthcare/medication needs, which then creates feelings of risk. One participant mentioned how they were unable to access their medication as they would sometimes be referred to nonprescribing clinicians:

I'm seeing a nurse practitioner on a Monday and they can prescribe and then two weeks later I'm seeing a nurse practitioner, but they can't prescribe ‘cause they've not done the training… (Patient 03, FG1)

### 3.3 Stage 2: Consensus workshops

A total of seven patients/carers (five patients, five carers, three had both roles) were recruited for workshop 1 and a total of seven healthcare professionals (three pharmacists, two GPs, two nurses) were recruited for workshop 2. There was a broad representation of ages in the patient/carer workshop, whereas in the healthcare professional workshop, the majority (*n* = 5) represented one age range bracket. Females comprised the majority (*n* = 5/7) in the patient/carer workshop and males comprised the majority (*n* = 5/7) in the healthcare professional workshop. Overall, across both workshops the most common ethnic group was White British (*n* = 10/14). In the healthcare professional workshop, 3/7 had a mental health role and 4/7 worked in a nonspecialist primary care role. A comprehensive breakdown of the patients/carers and healthcare professionals can be seen in Tables 2 and 3, respectively.

**Table 2:** Workshop 1—Patient/carer participants.

Patient/carer ID	Age range	Sex (M/F)	Ethnicity
Patient/carer 01	65+	M	White—any other background
Patient 02	65+	F	White British
Patient 03	25–34	F	White British
Patient/carer 04	55–64	F	White British
Carer 05	18–24	F	White British
Patient/carer 06	45–54	F	Mixed—any other background
Carer 07	55–64	M	Asian Indian

Abbreviations: F, female; M, male.

**Table 3:** Workshop 2—Healthcare professional participants.

Healthcare professional ID	Mental health role	Age range	Sex (M/F)	Ethnicity
Pharmacist 01	–	25–34	M	Asian Indian
Pharmacist 02	–	25–34	M	White British
Pharmacist 03	✓	25–34	M	White British
GP 04	–	25–34	F	White British
GP 05	–	25–34	M	White British
Nurse 06	✓	35–44	M	White British
Nurse 07	✓	35–44	F	White British

Abbreviations: F, female; GP, general practitioner; M, male.

A detailed summary of the themes generated and potential interventions proposed by workshop participants can be seen in Table 4.

**Table 4:** Themes generated from nominal group discussions.

Theme	Description	Participants proposed interventions
*Workshop 1—Patients/carers*		
Medicines use and support	▪ Timely access to correct medication. ▪ Reassurance regarding polypharmacy and side effects. ▪ Providing appropriate information about medications.	▪ Family and carer medication education. ▪ Expanded role of clinical pharmacists. ▪ Reassurance and advice regarding medication brand and supply issues. ▪ HCP education regarding neurodevelopmental disorders.
Service delivery, continuity and follow‐up	▪ Regular follow‐ups with familiar HCPs. ▪ Access to HCPs who can address medication needs. ▪ Services individually tailored to meet the needs of different mental health conditions.	▪ System alerts and role of clinical pharmacists. ▪ CPN assigned to each patient. ▪ Team approach including patient, carer and multiple healthcare disciplines. ▪ HCP education from lived experience experts.
Healthcare staff levels, skills, workload and communication	▪ Appropriate staff levels to meet demands. ▪ Adequate psychiatric training of HCPs. ▪ Communication between teams and across interfaces.	▪ Improved funding to increase staff retention and lower the workload. ▪ Peer support workers. ▪ Clear guidance and training for HCPs on how to manage and support patients with mental illness. ▪ Communication training. ▪ Connections with third sector and voluntary organisations.
Patient/carer empowerment and trust	▪ Power imbalances between HCPs and patients/carers. ▪ Lack of respect towards patients with mental illness. ▪ Lack of shared decision‐making.	▪ Promote shared decision‐making. ▪ Carer/family involvement.
Guidelines and resources	▪ Revision of guidelines to provide greater detail with patient/carer stakeholder input. ▪ Implementation, regular updates and electronic incorporation of detailed guidelines.	▪ Utilisation of expert patients and carers to assist with training and guideline development. ▪ Guidelines and updates communicated to HCPs and incorporated into prescribing systems.
*Workshop 2—Healthcare professionals*		
Patient interaction, support and holistic shared care	▪ Providing a holistic approach and exploring patient social factors. ▪ Lack of shared decision‐making. ▪ Patient access to additional resources in a timely manner.	▪ Increase appointment times. ▪ HCP education and training on communication with patients. ▪ Improve the quality of SMRs—conducted by an appropriate clinician.
Communication	▪ Communication between teams and across interfaces. ▪ Timely transfer of detailed clinical information. ▪ Ease of access to expert advice.	▪ Instant access to HCPs in relevant clinical teams. ▪ Dedicated phone lines to expert advice. ▪ Clinical system communication directly with psychiatric support. ▪ Mental health practitioner IPs in primary care.
Medication processes and standardised approach to care	▪ Increased access, time and follow‐up. ▪ Guidance on care responsibilities. ▪ Continuity of care and practice.	▪ Sharing good practice between community services—use of clusters.^a^ ▪ Detailed guidance on medications and responsibilities. ▪ Reminders/alerts for patients.
Healthcare staff knowledge and relationships	▪ Knowledge of mental health conditions, psychotropics and referral pathways. ▪ Cohesive working between teams and across different professional disciplines.	▪ HCP training with expert patients. ▪ Embed psychiatry and cohesive working throughout UG curriculum. ▪ Focused training on prevalent topics.
Culture and attitudes towards mental illness	▪ Dignity and respect during consultations. ▪ Stigma towards patients and dismissive of concerns.	▪ HCP education and training on how to communicate and support patients with mental illness.

Abbreviations: CPN, community psychiatric nurse; HCP, healthcare professional; IP, independent prescriber; SMR, structured medication review; UG, undergraduate.

a Groups of general practices in a close geographical location.

### 3.4 Ranking exercise

Following the discussion of ideas, theme generation and subsequent ranking exercise, participants were presented with the final group ranking to check the accuracy. The ordered ranking from highest to lowest priority for each workshop can be seen in Tables 5 and 6.

**Table 5:** Patient/carer ranked priority areas for interventions.

Theme	Rank 1 (score 5)	Rank 2 (score 4)	Rank 3 (score 3)	Rank 4 (score 2)	Rank 5 (score 1)	NGT score (7 participants)	Ranking order (highest to lowest priority)
Medicines use and support	3	3	0	1	0	29	1
Service delivery, continuity and follow‐up	2	2	2	0	1	25	2
Healthcare staff levels, skills, workload and communication	1	1	4	1	0	23	3
Patient/carer empowerment and trust	1	1	1	3	1	19	4
Guidelines and resources	0	0	0	2	5	9	5

Abbreviation: NGT, nominal group technique.

**Table 6:** Healthcare professionals' ranked priority areas for interventions.

Theme	Rank 1 (score 5)	Rank 2 (score 4)	Rank 3 (score 3)	Rank 4 (score 2)	Rank 5 (score 1)	NGT score (7 participants)	Ranking order (highest to lowest priority)
Patient interaction, support and holistic shared care	4	2	1	0	0	31	1
Communication	1	3	3	0	0	26	2
Medication processes and standardised approach to care	2	1	1	3	0	23	3
Healthcare staff knowledge and relationships	0	1	2	2	2	16	4
Culture and attitudes towards mental illness	0	0	0	2	5	9	5

Abbreviation: NGT, nominal group technique.

The ranked themes developed by all stakeholders within the workshops were used to summarise shared themes and highlight differences to help contextualise the rankings (presented in the following section).

### 3.5 Themes identified by patients, carers and healthcare professionals

#### 3.5.1 Patient support and holistic shared care

Patients and carers discussed the limited information they received regarding drug–drug interactions (polypharmacy), side effects, tapering regimens and therapeutic alternatives, all of which were reported to create anxiety. Having unclear instructions and untimely access to the correct medication was considered by patients/carers to exacerbate worries and create more concerns. Patients felt that family/carer support was important to identify and address these issues. Both patients/carers and healthcare professionals agree that providing timely education to carers, families and patients regarding insight into their mental illness, medication indications, side effects and tapering doses may help to encourage patients/carers to take an active role with their medication, provide reassurance, prevent issues from occurring with medication and promote shared decision‐making. Healthcare professionals also discussed the importance of adopting a holistic approach to improving care for this patient group. Clinicians placed particular emphasis on the elevation of social prescribing and a need to reduce unnecessary prescribing of psychotropics (i.e., deprescribing). They felt that professionals adopting a ‘checklist’ approach (i.e., performing tasks following sequential criteria) impacted the consideration of holistic needs of the patient (see File S3 for detailed quotes of themes from each consensus workshop).

#### 3.5.2 Healthcare professional workload, communication and skills

Ensuring appropriate staffing levels with manageable workloads were highlighted by patients and carers as important areas for improvement. Patients/carers reported observing an increased workload for services which they felt adversely affected time to prescribe and communicate effectively, as well as staff retention issues which may result in outsourcing to agency staff. However, patients/carers acknowledged that to achieve improvement in this area, it may require wider system/policy changes.

Consistency of communication was highlighted by all stakeholders and communicating accurate information to patients was discussed by healthcare professionals, caused by absent or untimely transfer of brief clinical information between teams and across interfaces. Clinicians felt that being able to have quick and easy access to expert advice would help support the prescribing, monitoring and amendments of psychotropic medications; as it was reported that handover/discharge documents may be unclear where clinical responsibility for the patient lies, which can cause confusion around how professionals should act. Healthcare professionals highlighted a need for better working relationships between primary and secondary care teams, and that ensuring a parity of skills within the workforce may foster respect within and across professional disciplines.

There was agreement from patients/carers and healthcare professionals that both clear guidance/training regarding mental health conditions and communication were imperative. Healthcare professionals also felt it was an important goal to increase the knowledge, skills and competence of primary care clinicians regarding psychotropics, mental illnesses and referral pathways. Improved training was acknowledged as a potential solution; however, healthcare professionals stressed a need for memorable teaching (e.g., use of expert patients) to ensure professionals are not overloaded with regular ‘checkbox’ training exercises, which were considered to have poor engagement from professionals.

#### 3.5.3 Service delivery, continuity and follow‐up

Patients/carers and healthcare professionals discussed the need for stability within the patient healthcare journey by having timely follow‐ups with familiar healthcare professionals. Following this approach was reported to help with continuity of care and helped to ensure regular reviews with the appropriately skilled clinician. The absence of a professional/key worker taking responsibility for patient care made patients/carers feel ignored and receiving insufficient support. Holistic approaches to care via a collaborative effort between patients, carers and various healthcare professionals were highlighted as a potential solution. Healthcare professionals felt that a standardised approach to consultations with all‐encompassing guidelines, which outline clear guidance on clinical responsibility, could help achieve the same standard of care for this patient group. Whilst acknowledging the importance of uniformity of care, they also recognised the importance of the individuality of each patient, and that to achieve this outcome, key stakeholder involvement would be needed to ensure adaptability of any interventions.

#### 3.5.4 Culture and patient empowerment

Attitudes towards patients with mental illness and the related stigma of mental health diagnoses was raised by both patients/carers and healthcare professionals. It was perceived by all stakeholders that patients with mental illness were not receiving the same level of respect and dignity as other patient populations. Healthcare professionals were mindful that this can translate into dismissing patient concerns and reported observing a culture of negative attitudes towards this patient group within healthcare and felt that these attitudes needed to be addressed to tackle this issue. Patients and carers felt a lack of respect towards them from healthcare professionals which affected trust, power dynamics and patient/carer empowerment. Patients and carers discussed the importance of building therapeutic relationships, which were considered to help instill trust with healthcare professionals. Patients and carers emphasised that equal recognition of both their and healthcare professionals' opinions was instrumental in supporting an effective care journey, promoting patient/carer empowerment and ensuring better care.

#### 3.5.5 Guidelines and resources

Patients/carers solely discussed this theme and highlighted that important considerations for change included the updating and implementation of clinical guidelines, as well as strong working relationships between healthcare professionals and third‐sector support groups. The importance of funding for the provision of support groups was raised, and the need to be mindful that the availability of this resource can fluctuate between regions. Patients/carers placed additional emphasis on the development of patient‐centred guidelines and the need to ensure updates are communicated swiftly to healthcare professionals. They felt that the latest clinical guideline updates being incorporated into primary care electronic prescribing systems could allow their healthcare providers to optimise the prescribing of medication more effectively.

## 4 DISCUSSION

This study has highlighted multiple factors contributing to how safe patients with mental illness and their carers feel with their medication in primary care. The findings from this study build upon previous research ^11^ to construct a holistic picture of factors which contribute to medication safety incidents affecting this patient group. These factors were then used to inform stakeholder‐led, nominal group consensus workshops to identify priority areas for interventions. There was considerable overlap between the themes generated from both patient/carer and healthcare professional workshops, with the main difference being the presence of guidelines and resources as a theme in the patient/carer workshop. Key areas of mutual agreement for interventions included communication, patient support with holistic continuity of care and follow‐up, shared decision‐making and empowerment, staff knowledge and workplace culture, patient dignity and respect. Previous research examining priority improvement areas for medication safety in primary care amongst the general population, found communication between services, inappropriate reviews and patient‐related factors as key safety concerns.^41^ It was noted that solutions should focus on patient education, unified digital systems and improving communication.^41^ This study supports this focus; however, attention must also be paid to more unique challenges identified in this study such as healthcare professional mental health knowledge, shared decision‐making, culture and patient respect. Patient and carer involvement in mental healthcare safety has often been impeded by service user fears of repercussions; concerns about being ignored by services; and difficult processes for raising safety concerns.^42^ Patients have been viewed as a contributory factor to safety incidents, whereas this study highlights how this patient group can provide useful insight into how to resolve patient safety issues.^43^ This study highlights the importance of ensuring that patient/carer experiences are considered when assessing and improving quality of healthcare services and supports the active involvement of patients and carers in healthcare safety research; an approach which is increasingly being encouraged.^23^, ^24^

Communication across care interfaces, between professionals and from professional to patient was highlighted as an important issue. The healthcare professional perspective study ^11^ highlighted that there may be absent or only brief clinical information communicated between professionals which can lead to uncertainty in how to respond. Previous research has highlighted that limited clinical information is common in primary care and can lead to adverse patient outcomes.^44^ Comprehensive integration of electronic health records has been reported to strengthen relationships between clinical providers and enhance primary care outcomes, including medication safety and cost‐effectiveness.^45^, ^46^ There is a clear need for a unified digital service which allows interoperability and access by clinicians from within both primary and secondary mental health services. Participants highlighted a need to have instant access to relevant clinical information to help address communication challenges between clinical teams and across care interfaces. Future work should now investigate the feasibility of a unified, interoperable digital system across the primary‐secondary care interface to facilitate this flow of clinical information. Despite communication issues between professionals and patients, relatively few interventions have been trialled to structure mental health consultations in primary care, even though feasibility of interventions has been demonstrated.^47^ With other studies noting communication issues within healthcare,^48^, ^49^ it is important to address this issue given the agreement of both patients/carers and healthcare professionals about its importance.

Patients/carers highlighted a lack of empowerment with their treatment decisions and what they perceived to be an authoritarian approach taken by clinicians, as issues that affect their feelings of safety and which impact shared decision‐making. Shared decision‐making has multiple benefits including increased satisfaction with care/services and greater patient engagement and better patient outcomes.^50^, ^51^ Despite being the recommended approach in psychiatry,^52^, ^53^, ^54^, ^55^ use of this model may be limited in comparison to its use in physical healthcare.^56^, ^57^, ^58^ Previous research has already established that a power imbalance may exist between healthcare professionals and patients with mental illness.^34^, ^35^ It may be that empowerment is more of a key issue in the mental health context, as patients experience increased stigma in healthcare.^59^ Patients/carers highlighted how they may have little input into medication choice, as well as being provided with little information about treatment risks. They also highlighted how this can lead them to undertake decisions about medication without informing the clinician, which can increase the risks of harm. Since previous research has identified important barriers to shared decision‐making in mental healthcare such as clinician concerns and engagement,^60^ it is important that future research finds a way to overcome these barriers to effectively implement this model into routine practice.

Limited knowledge regarding psychotropics and mental illnesses amongst healthcare professionals was identified as an important issue and a key area for improvement interventions, and this finding is consistent with previous research.^61^, ^62^, ^63^, ^64^, ^65^, ^66^, ^67^, ^68^ Since this is a clearly defined barrier to the safe provision of primary care mental health services, educational and health service reform is now required. In England, there is an agenda to bring more mental health specialist services into primary care,^2^ which may help address the knowledge gaps. Clinical pharmacists could also be key in responding to knowledge and continuity issues, as they have strong foundational knowledge regarding neuropharmacology/therapeutics and could provide regular clinical follow‐ups.^10^, ^11^, ^69^, ^70^, ^71^, ^72^ With pharmacists in England being trained as ‘prescriber‐ready’ at the point of registration from 2026,^73^ this would be an opportune moment to explore how their skills may be developed to address these key issues in primary psychiatric care.

### 4.1 Strengths and limitations

This study used a well‐known standardised approach to obtaining consensus in health services research ^28^, ^74^ and by doing this it enabled balanced input from all participants, without pressure to conform.^30^ The inclusion of patients, carers as well as healthcare professionals helps ensure the interventions suggested addressed the needs and priorities of everyone within primary healthcare services.^15^ FGs and consensus workshops were conducted solely online, so it is acknowledged there may have been an element of digital exclusion^75^; however, this also enabled greater national reach to participants who may have otherwise been inaccessible.^76^ Due to participants self‐selecting into the study, it may therefore be difficult to generalise these findings to a large population.^77^ Conducting the patient/carer workshop first and providing their ranking to healthcare professionals enabled the research to be patient/carer led, and enabled healthcare professionals to consider the needs of patients more deeply. However, this approach may have increased the likelihood of the professionals identifying similar issues at the expense of other issues, especially given the overlap in findings from both workshops. There was a proportionate representation from different healthcare disciplines and mental health roles, however, there was no representation from psychiatrists which may mean the interventions do not consider the needs of all key stakeholders within these services. It is known that ethnicity is linked to disparities in mental healthcare,^78^, ^79^, ^80^ but as most participants in this study were from one ethnic group, these findings may not reflect the experience of those from other ethnic groups.

## 5 CONCLUSION

This is the first study to explore the factors that make patients with mental illness and their carers in primary care feel safe/unsafe with their medications, and to identify priority areas to target for intervention using key stakeholder input. The findings were that issues around lack of empowerment, respect, communication and knowledge reduced a patient's feelings of safety with medication in primary care and these were identified as key areas to target for intervention. To develop interventions that consider the needs of all key stakeholders, it is important to consider multifaceted approaches, since the causes of medication safety challenges for this patient group are multifactorial.

## AUTHOR CONTRIBUTIONS

**Matthew J. Ayre**: Conceptualisation; investigation; writing—original draft; methodology; validation; writing—review and editing; formal analysis; data curation; visualisation. **Penny J. Lewis**: Conceptualisation; investigation; methodology; formal analysis; supervision; writing—review and editing; validation. **Denham L. Phipps**: Conceptualisation; investigation; methodology; validation; writing—review and editing; formal analysis; supervision. **Kathy M. Morgan**: Writing—review and editing; validation; formal analysis. **Richard N. Keers**: Conceptualisation; investigation; methodology; validation; writing—review and editing; formal analysis; supervision.

## CONFLICT OF INTEREST STATEMENT

The authors declare no conflict of interest.

## ETHICS STATEMENT

This study was approved by the University of Manchester Research Ethics Committee 3 (reference 2023‐16161). Participants provided their written informed consent to participate in this study. Written informed consent was obtained for the publication of data in an anonymous format as included in this article.

## Supporting information

Supporting information.

Supporting information.

Supporting information.

## Data Availability

The data generated and analysed during this study to support the findings are included in this published article and its supplementary files.
