# Supplementary material for: Towards understanding and improving medication safety for patients with mental illness in primary care: A multimethod study
Source: Health Expect. 2024 May 30;27(3):e14095. doi: 10.1111/hex.14095 (PMC11139968; doi:10.1111/hex.14095)
Supplement: Supplementary file 1 — Supporting information. [file HEX-27-e14095-s003.docx]

**Focus group schedule**

**Background**

In our previous work we have talked to healthcare professionals about the factors they thought contributed to risk with medicines for patients with mental illness outside of hospital. We would now like to explore what factors are important from a patient and carer perspective which will help us develop a more complete picture of the safe use of medicines outside of hospital.

The aim of this focus group is to get your views about what makes you feel safe and unsafe with your medicines outside of hospital. This includes any factors that you think are important during these occasions where you might feel more or less safe, and what patients, carers and healthcare professionals can do to increase the safe use of medicines.

**Ground rules**

1. This discussion is strictly confidential, please ensure you are in a private space and unlikely to be disrupted.
2. The session is being audio/video recorded using Zoom, so speak loud/clearly and do not speak over one another. Please wait for someone to finish their point before stating yours.
3. Please respect each others right to express views/opinions, even if it differs from your own.
4. We will anonymise the transcripts so nobody can be identified by name. However, please try to avoid naming buildings/services/patients/healthcare professionals. If anyone accidentally mentions any names then please can everyone treat that information with strict confidentiality.

**Questions**

1. Can you describe what makes you feel safe (or unsafe) with your medicines outside of hospital? You can talk generally or talk about specific occasions if you wish.
2. How do these things lead to you feeling safer/less safe with your medicines?
3. What might need to happen or change for you to feel more safe (less safe) with your medicines outside of hospital? You can talk generally or talk about specific occasions if you wish.
4. Are there any issues that you wish to discuss?

**Prompts**

- Can you tell me more about that?
- X person said this, what is your view on it?
- Why have you said that?
- X person had a different view to Y, can you tell us more about your views?
