# Supplementary material for: Towards understanding and improving medication safety for patients with mental illness in primary care: A multimethod study
Source: Health Expect. 2024 May 30;27(3):e14095. doi: 10.1111/hex.14095 (PMC11139968; doi:10.1111/hex.14095)
Supplement: Supplementary file 2 — Supporting information. [file HEX-27-e14095-s002.docx]

**Quotes to supplement the stage one focus group themes**

| **Focus group theme** | **Quote** |
| --- | --- |
| Communication | *“…the communication aspect isn't great because you're in-between different professionals. And if you've got any kind of mental health troubles, being in that situation can exacerbate your symptoms…” (Patient/carer 10, FG3)* |
| Trust, involvement and respect | *“Nobody knows what they're doing, do they. […] your doctor doesn’t know what the psychiatrist is doing, the psychiatrist […] suggests services that aren't appropriate for you or gives you tablets that…I mean, ‘cause I had a kidney failure, I had sepsis, and he suggested a tablet that was contraindicated for […] impaired renal function. And […] I just don’t have confidence in the medical professionals to look after me properly.” (Patient/carer 07, FG2)*  *“I feel would they be talking to me the same way, would they be treating me the same way if they didn't know that I was on this medication and know what it's for? And that does feel quite unfair sometimes. And I do feel like it can feel quite alienating.” (Patient 09, FG3)* |
| Continuity and support | *“I've never had any experience of having a follow-up for the medication. I've kind of tried different medications and then found that medications have not suited me for different reasons. But it's been more me as a patient asking to see somebody and talking about side effects…” (Patient/carer 10, FG3)*  *“I can understand why you could almost become reluctant to take the medication you’ve just been prescribed ‘cause […] I think with most drugs if you look hard enough, you could find someone with a horror story and I feel like having resources where people could look and get factual information […] might be quite useful.” (Patient 06, FG2)* |
| Access | *“…the right medication delivered on time, the right quantity, the right dosage. So that’s safety. If that fails, that’s when risks start to appear. And I have to chase people. And there’s a risk of [the patient] not taking her medication, which can then cause a relapse.” (Carer 02, FG1)* |
| The Patient and Carer | *“…there's more effort being made, […] an onus on the patient rather than there being a bit more responsibility from the prescriber… (Patient/carer 10, FG3)*  *“I would know absolutely nothing about medication and things like that. So a lot of things I have to ‘Google’ and say, is this normal if […] it’s a new medication, is it normal to maybe have a small reaction while your body gets used to it or things like that.” (Carer 01, FG1)* |
