# Supplementary material for: Towards understanding and improving medication safety for patients with mental illness in primary care: A multimethod study
Source: Health Expect. 2024 May 30;27(3):e14095. doi: 10.1111/hex.14095 (PMC11139968; doi:10.1111/hex.14095)
Supplement: Supplementary file 3 — Supporting information. [file HEX-27-e14095-s001.docx]

**Quotes to supplement the stage two consensus workshop themes**

| **Patient/carer workshop theme** | **Quote** |
| --- | --- |
| Medicines use and support | *“It should be clear that when you have a first consultation or even consultations going on from that, that you should be told why they're going to put you on that medication. […] what it is about that medication that suits your needs, and being really clear about any side effects and really clearly signposting where you go from that. So if you do get any of these side effects don't worry, […] this is where you go with that. If this medication doesn't work for you don't worry, there are alternatives.” (Patient 03)* |
| Service delivery, continuity and follow-up | *“It's about having that discussion as a team of different people. So the pharmacist, the patient, the carer, somebody else, do people know about advocacy support? There needs to be like a little team of people involved, rather than it just being all separate in some ways.” (Patient/carer 06)* |
| Healthcare staff levels, skills, workload and communication | *“The workload is an issue. So retaining staff on a permanent basis, […] less workload and better pay and better conditions, then they [healthcare providers] wouldn't need to get locums and pay silly money to locums, and patients would get continuity of care instead of seeing different people every time…” (Carer 07)* |
| Patient/carer empowerment and trust | *“Health professionals do not regard the carers as an equal and they should do for that person that they're caring for. Because they [the carer] know the whole history of that person.” (Patient/carer 01)* |
| Guidelines and resources | *“I don't know that information [the prescriber] has got in front of them is current and updated based on the guidelines produced say last week. What is the timeline between a new guideline being produced and that being incorporated into the computer system…” (Carer 07)* |

| **Healthcare professional workshop theme** | **Quote** |
| --- | --- |
| Patient interaction, support and holistic shared care | *“Quite often, when patients come in for these reviews, it's a very brief […] how are you doing, whatever. And then it's […] have you ticked all the boxes that you need to tick. And there's maybe not as much focus, actually, on the patient, and their mental illness, and it's more just trying to* *[…] it's almost like a means to an end.” (Pharmacist 02)* |
| Communication | *“…with very little communication, in terms of what's been prescribed, and how that’s going to be managed. […] especially if you need to optimise doses, or de-prescribe it. And at any point, the information that comes out is terrible, in general.” (Nurse 07)* |
| Medication processes and standardised approach to care | *“I think it would be useful to look in more detail, as to what people are actually doing. So, if you have a guideline around depression, anxiety, and any other […] mental health condition, […] you would need to ask the GPs, […] the nurse practitioners, and everyone else who works with these patients, what are you actually doing, how are you interpreting this, what's happening within your area. […] I think it would need to be an adaptable intervention, that practices can […] fit into their context. Because, naturally, that’s what's going to need to happen in order for it to work most effectively.” (GP 05)* |
| Healthcare staff knowledge and relationships | *“So this is obviously one topic of, however many hundreds of topics that GPs need to be aware of. So, as soon as we start suggesting doing regular training, another speciality would make a similar suggestion, that regular training needs to be done for whatever they consider to be most important, related to their context. I think, whatever the intervention is, if training is a suggested solution, it does maybe need to be a little bit more substantial, so it has some longevity, so it doesn't need to be as regular.”  (GP 05)* |
| Culture and attitudes towards mental illness | *“Change the culture, treat it as, […] have awareness campaigns, treat it as the same as, […] mental health gets treated the same way as you would do for physical health problems.” (Pharmacist 03)* |
